# Construction of metal endocrown for a short clinical second molar crown

**DOI:** 10.1002/ccr3.8522

**Published:** 2024-02-07

**Authors:** Saad AlHazzani, Entessar AlAnazi, Abdulrhman AlMazam

**Affiliations:** ^1^ Saudi Board in Restorative Dentistry, Dental Department King Khalid Hospital AlKharj Saudi Arabia

**Keywords:** endocrown, endodontically treated tooth, metal endocrown, post‐endodontic restoration, second molar, short crown

## Abstract

This case highlights where a metal endocrown was used as a novel technique. The metal endocrown showed promising outcomes during the 5‐year follow‐up, utilizing a resin base to preserve tissue and act as a stress breaker. This approach was used to preserve the tooth and avoid extraction or periodontal surgery.

## 1 INTRODUCTION

Endocrown is one of the options for coronal coverage of endodontically treated teeth in cases with limitations such as extensive loss of coronal structure or limited interocclusal space, which may pose challenges for dental specialists.^1^, ^2^ These crowns were first described by Bindl & Mörmann, who based their designs on the concept of a monolithic ceramic restoration developed by Pissis.^1^, ^2^

Endocrowns are a good treatment option for teeth with extensive loss of coronal structure and compromised structural integrity, which have a high risk of biomechanical failure, and are particularly challenging.^3^, ^4^, ^5^, ^6^, ^7^ They are cemented into pulpless teeth, fitting into the internal part of the pulp chamber for stability and retention. Endocrowns cover all crown margins and cusps, eliminating the need for intraradicular anchorage.^8^, ^9^

Endocrowns offer advantages such as being a conservative technique, preserving tooth structure, and reducing the need for extensive tooth preparation associated with conventional crowns. They are also less time‐consuming and require fewer dental visits.^10^, ^11^

Although this is a relatively new technique, reports already exist in the literature describing new materials and techniques for the construction of endocrowns; BioHPP (high‐performance polymer) is a material used in prosthetic dentistry known for its elasticity and stress‐distributing characteristics. Certainly, when dealing with a short clinical crown (limited exposed tooth structure), BioHPP's elasticity and stress distribution characteristics can be advantageous. The material's properties may aid in providing a secure and well‐fitting crown despite the challenges posed by a shorter tooth structure. This can contribute to the stability and longevity of the prosthetic restoration in such cases.^12^

Research has reported on various materials and technique used to fabricate endocrowns. Zoidis et al used a modified polyetheretherketone (PEEK) framework material. The unique property highlighted is the elastic modulus of PEEK, which is noted to offer improved protection to the tooth structure against occlusal forces compared with ceramic materials. This choice seems to be based on the material's ability to absorb and distribute occlusal forces more effectively, potentially contributing to the success and longevity of the endocrown. The use of PEEK combined with indirect light‐polymerized composite resin in the veneering process may represent a strategy aimed at enhancing the restoration's resilience under occlusal stresses compared with other approaches by using ceramic restorations.^13^

This case report is based on a novel technique described by Dr. AlHazzani, which explores new techniques and materials for constructing endocrowns in short interocclusal spaces of endodontically treated teeth.^14^ The technique was also utilized by Mittal et al. in their case report.^15^

This case report describes the use of a metal endocrown as a novel technique in a severely limited interocclusal space and crown lengthening contraindicated.

## 2 CASE REPORT

**FIGURE 1:**
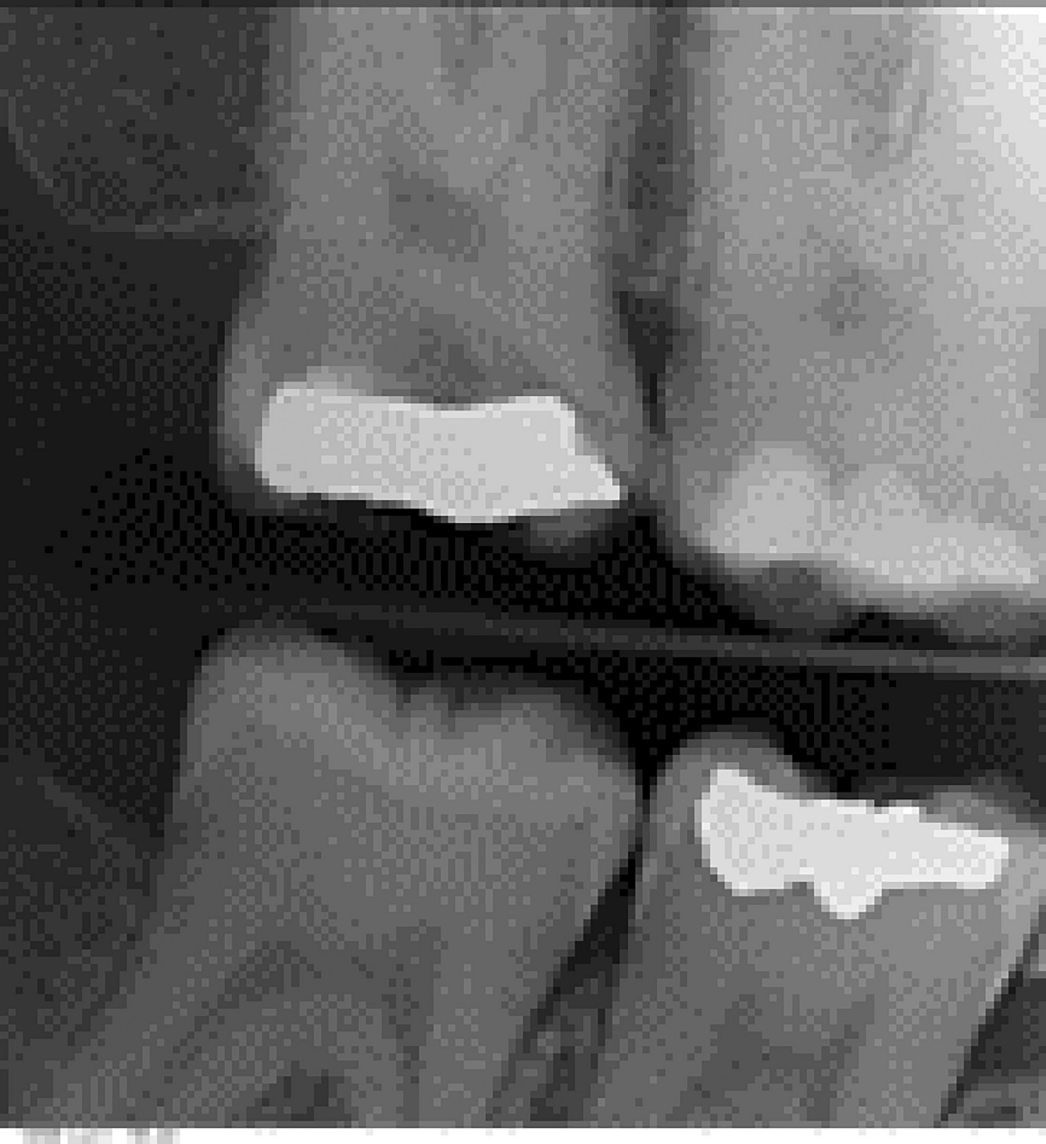
Preoperative periapical radiograph.

**FIGURE 2:**
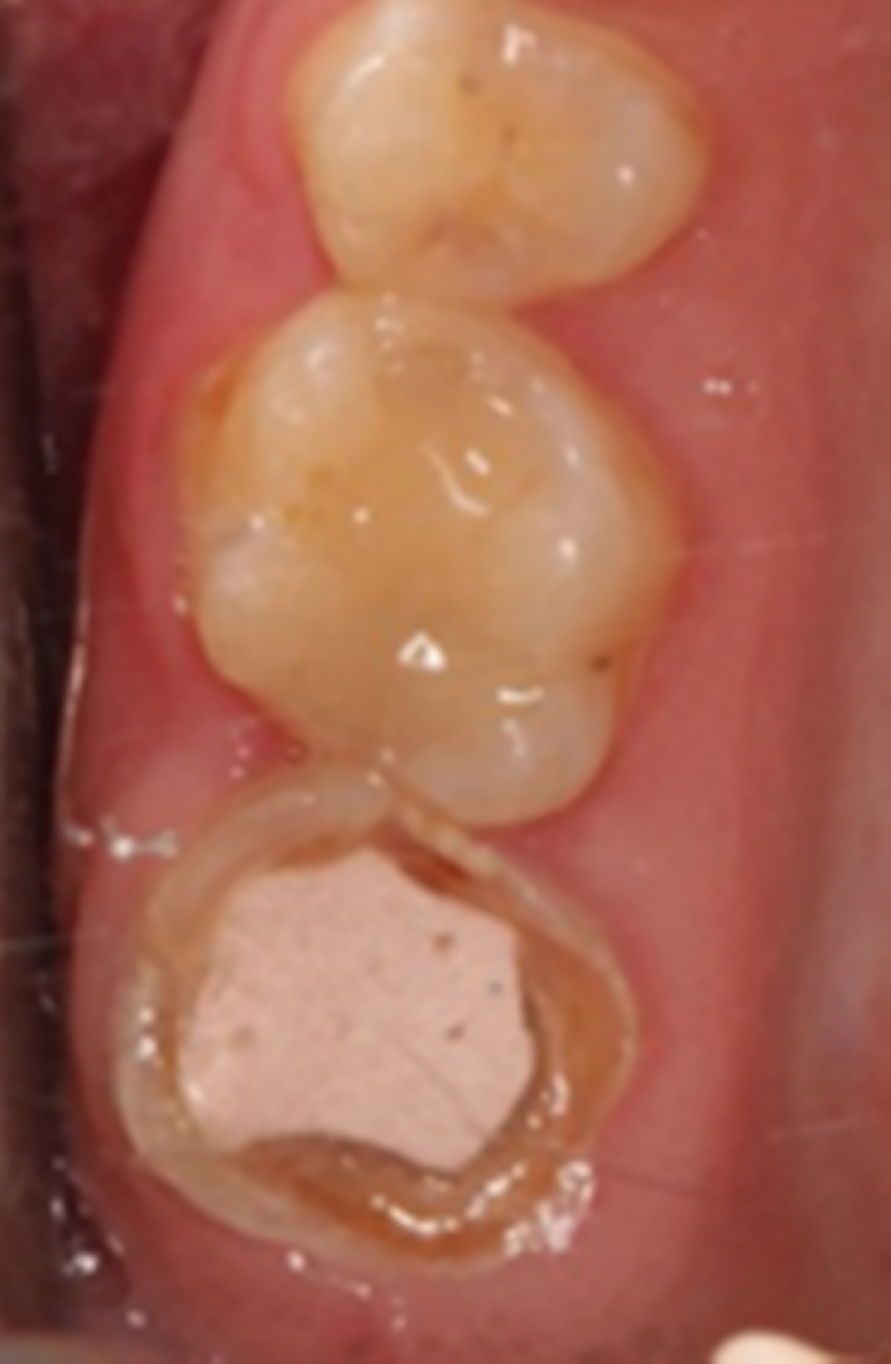
Clinical occlusal view of tooth 17.

**FIGURE 3:**
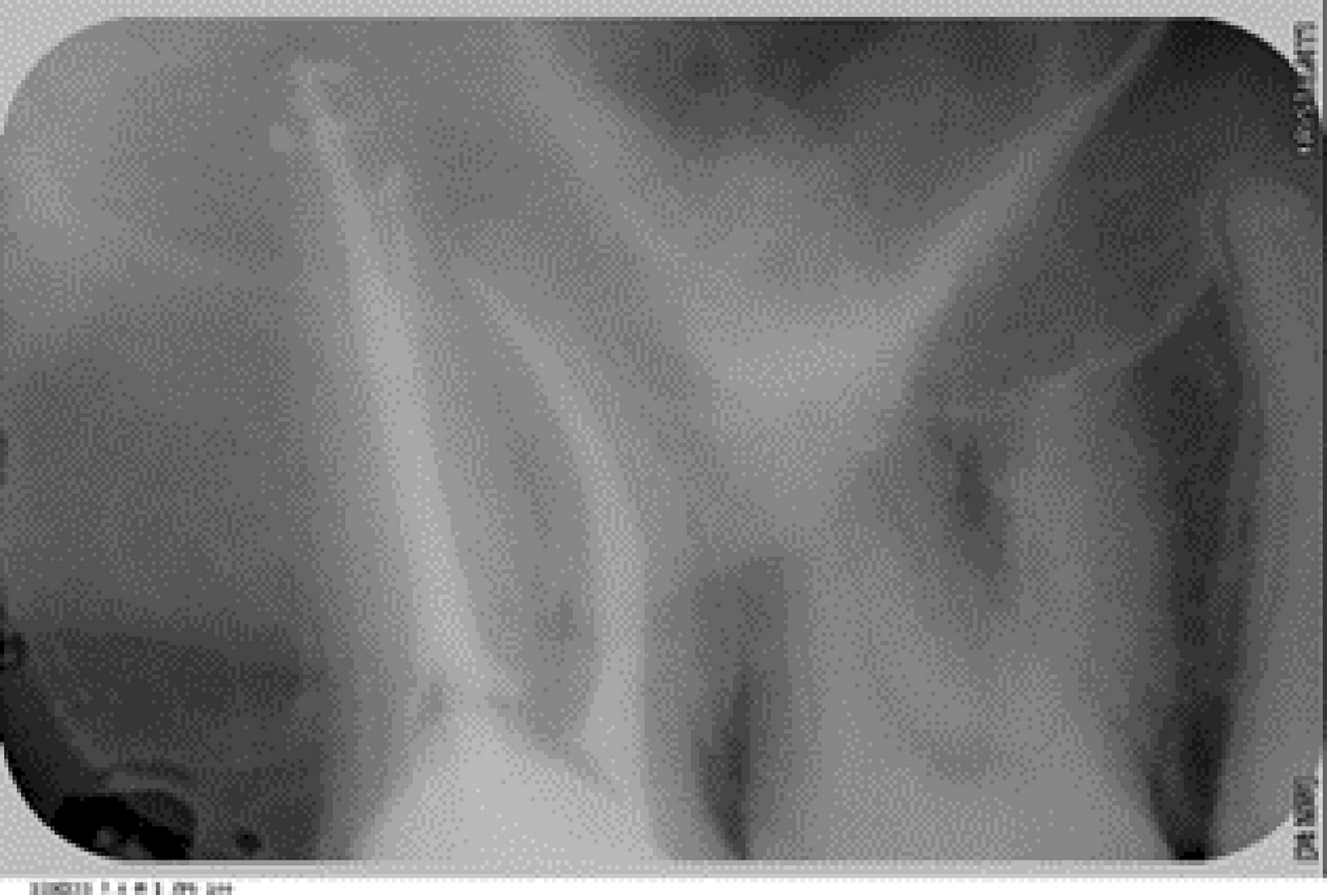
PA radiograph following root canal retreatment, tooth 17.

A 24‐year‐old Saudi woman, with no known medical conditions, visited our restorative dental clinic. She was referred by a general dental practitioner who had performed pulp extirpation and placed a temporary filling for her upper right second molar (tooth 17), which was diagnosed with deep caries and irreversible pulpitis (Figure 1). Clinical examination showed a baldly destructed tooth with a temporary filling placed (Figure 2). Endodontic treatment was performed successfully for the root canals of tooth 17 (Figure 3).

Due to inadequate interocclusal space for a single crown, treatment options were discussed with the patient. The technique of fabricating a metal endocrown (AlHazzani technique),^14^ indicated for cases with severely short clinical crowns like this one, was chosen based on its promising results and success.

**FIGURE 4:**
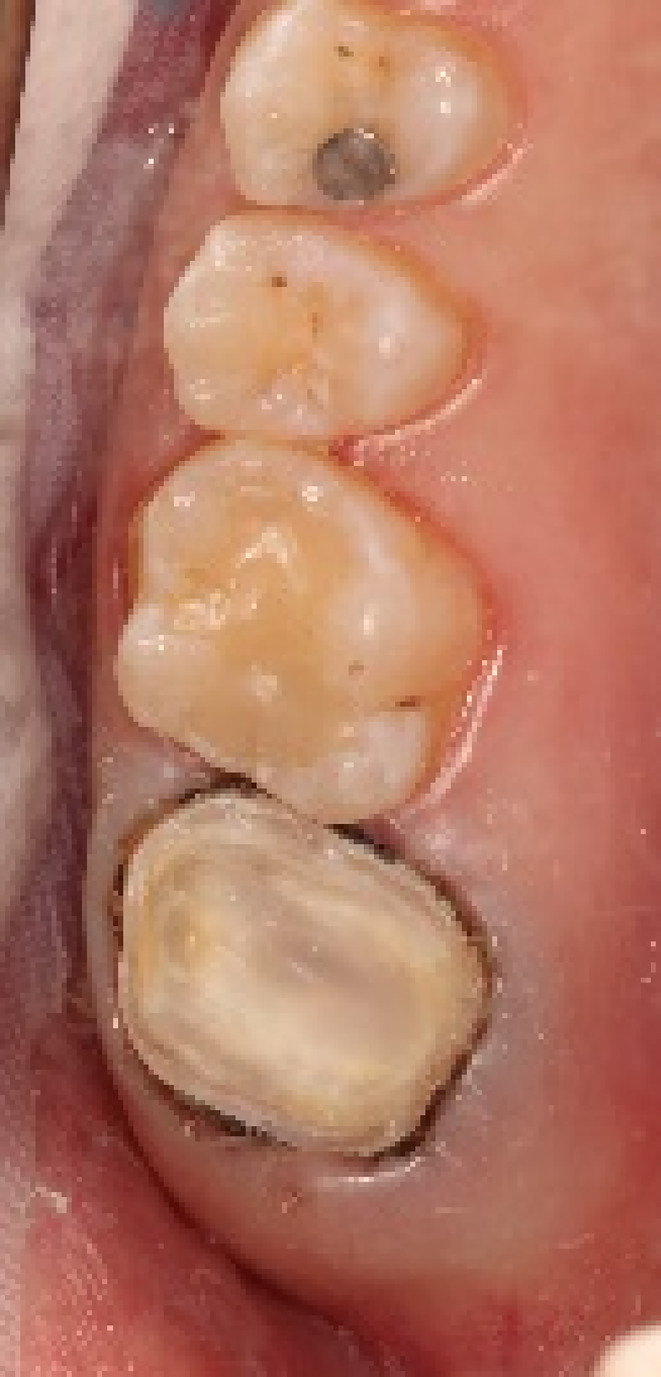
Preparation for metal endocrown on tooth 17.

After removing the temporary filling, the cavity and interocclusal space were evaluated. Tooth preparation for an endocrown consists of a circumferential from 1.0‐ to 1.2‐mm butt‐joint margin and a central cavity inside the pulp chamber for retention. Asupracervical flat butt joint is considered the best margin design for better stress distribution. Additionally, this results in a less traumatic procedure, preserving the marginal periodontium and facilitating impression procedures (Figure 4).^15^

**FIGURE 5:**
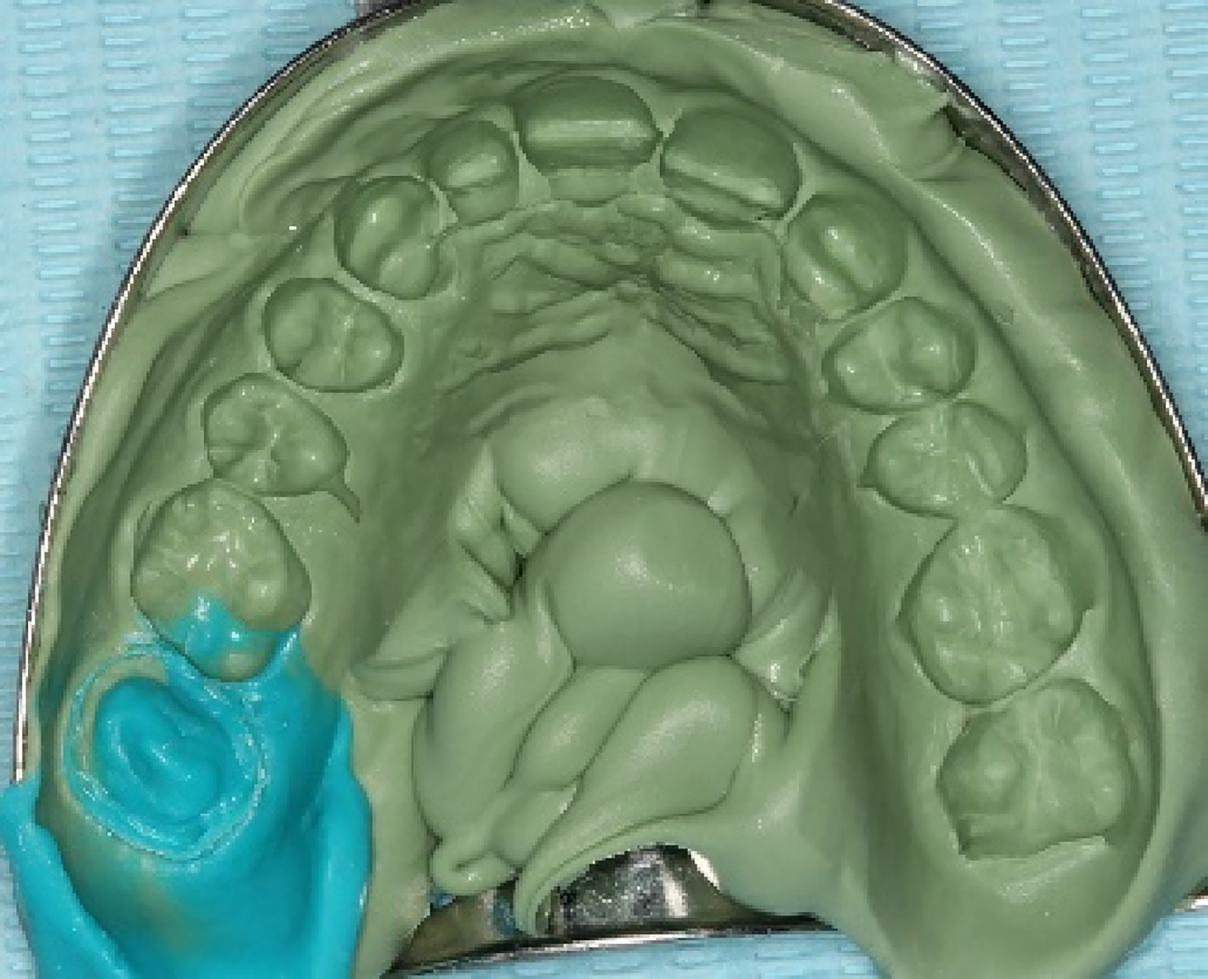
Impression for tooth 17.

Retraction cords were placed, and the cavity was etched with Ultra‐Etch® (Ultradent Products Inc., South Jordan, UT, USA). Single Bond Universal Adhesive (3M ESPE, St. Paul, MN, USA) was applied before the flowable composite (Filtek™ Z350 XT, Shade A3, 3M ESPE, St. Paul, MN, USA) was placed on the floor of the pulp chamber. An impression was obtained using retraction cords and vinyl polysiloxane (Aquasil Ultra Rigid, regular set, DENTSPLY Caulk, Milford, DE, USA). Both heavy and light body consistencies were used. An impression was taken using retraction cords and vinyl polysiloxane (Aquasil Ultra Rigid, regular set, DENTSPLY Caulk, Milford, DE, USA) in both heavy and light body consistencies (Figure 5). A provisional restoration was fabricated using bisacryl resin (Protemp™ 4, Shade A2, 3M ESPE, Neuss, Germany).

The endocrown was then fabricated using a noble metal dental alloy (PX Bond SF, PX Dental SA, Marin, Switzerland). The alloy composition included gold (Au) 51.2%, palladium (Pd) 38.6%, indium (In) 8.6%, gallium (Ga) 1.5%, and ruthenium (Ru) <1%.

**FIGURE 6:**
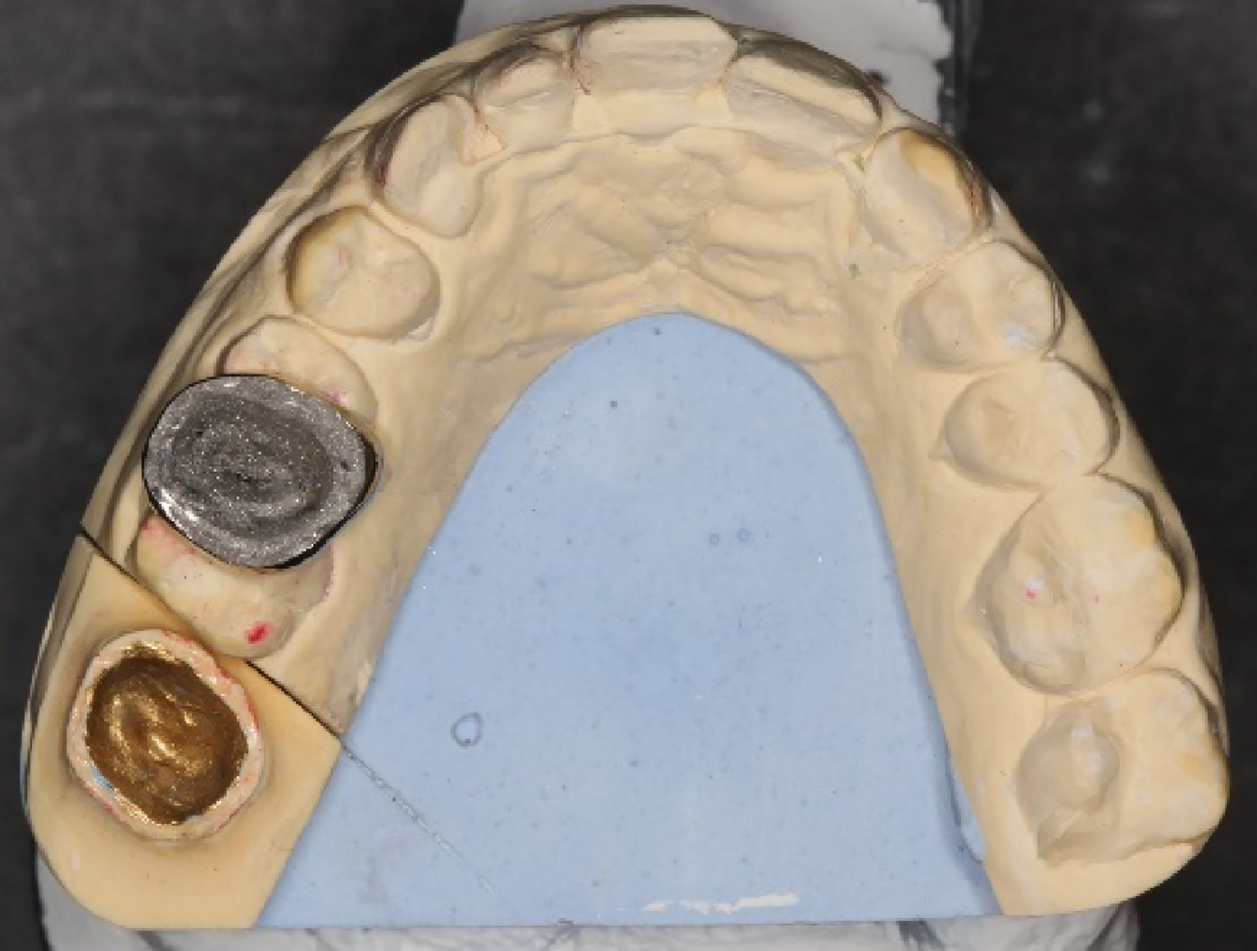
Sandblasted internal surface of the noble metal endocrown with the master cast.

After the try‐in step, the metal endocrown was sent to the laboratory for sandblasting the internal surface using a sandblaster (Basic Classic sandblaster, Renfert GmbH, Hilzingen, Germany) (Figure 6).

The endocrown restoration was bonded with a self‐adhesive universal resin cement (RelyX™ Unicem, 3M ESPE, St. Paul, MN, USA). Excess cement was removed, and the restoration was light‐polymerized using an LED curing unit (LITEX™ 695 C, DENTAMERICA®, CA, USA) for 40 s per margin. Any occlusal interference was checked and adjusted using metal finishing instruments.

## 3 RESULT

**FIGURE 7:**
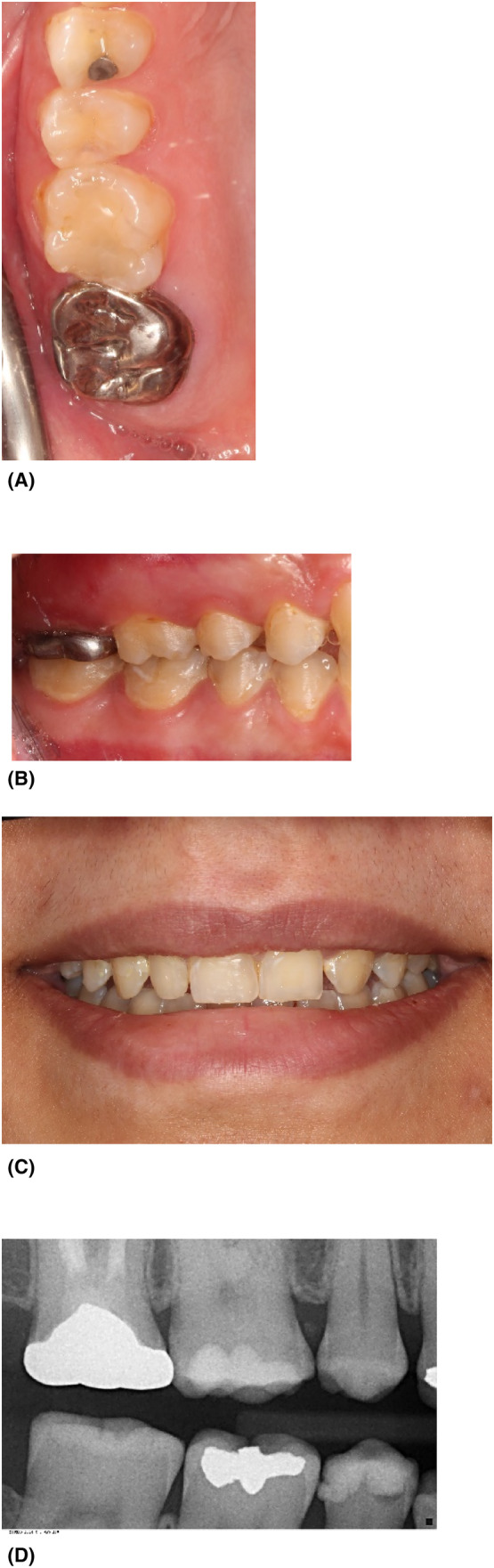
Final results (A), lateral view (B), patient smile (C), bitewing radiograph after cementation.

The final restoration is shown in Figure 7. These images demonstrate the successful restorative outcome The final restoration showcases optimal contour and seamless integration.

**FIGURE 8:**
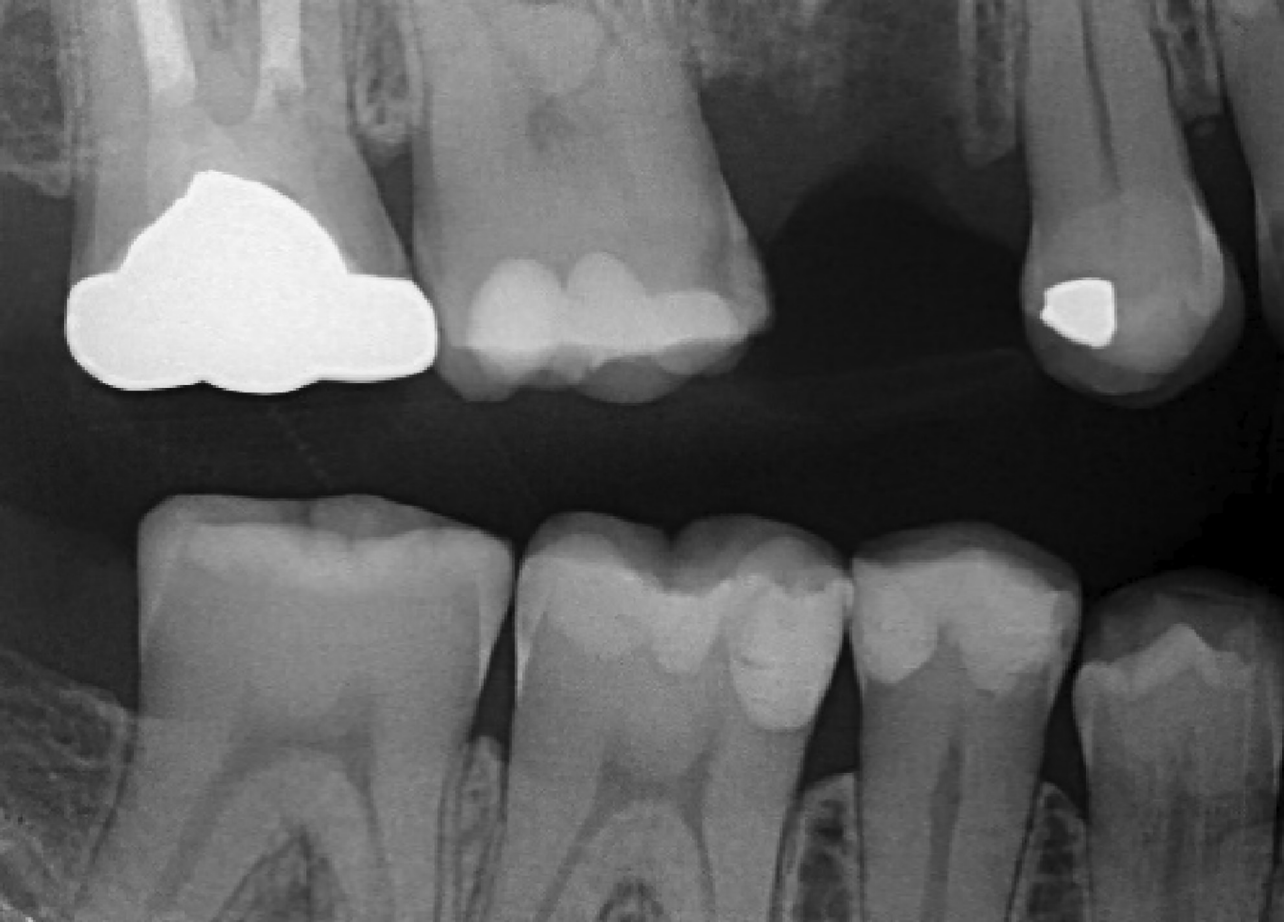
Bitewing radiograph at 5‐year follow‐up.

The follow‐up visit after 5 years confirms the stability of the restoration with no signs of recurrent caries or periapical issues (Figure 8). These images illustrate the effective management of the case, highlighting clinical excellence and a satisfying, enduring restorative outcome.

## 4 DISCUSSION

The metal endocrown is a dental restoration technique that has gained attention in recent years due to its promising longevity and effectiveness, as indicated by various in vitro studies available in the literature. One of the advantages of metal endocrowns is the reduced adhesive interface failure, which can be attributed to the decreased effect of multiple interfaces in endocrowns compared with other restoration techniques.

To ensure better stress distribution and preserve the marginal periodontium, it is recommended to have a supra‐cervical flat margin with a butt joint. This margin design not only contributes to improved stress distribution but also facilitates the impression‐taking process and results in a less traumatic procedure.

Different materials and techniques have been reported for fabricating endocrowns. The initial description of endocrowns involved the use of the CAD‐CAM system, specifically the CEREC system by Sirona. Silicate‐based ceramics were predominantly used with CAD‐CAM systems for crown fabrication.

In terms of fracture resistance, most reports indicate that metal endocrowns have higher fracture resistance compared with post‐core supported ceramic crowns. This increased fracture resistance is attributed to the greater thickness of metal endocrowns occlusally. However, there are some conflicting reports that suggest ceramic endocrowns do not provide any advantages over conventional crowns in terms of fracture strength.^16^

In an attempt to address the strength issue, some studies have explored modifying the materials used for fabricating endocrowns. These attempts have involved using resin composites or modified ceramics with other compositions such as zirconia and polymers. For example, one study reported higher fracture resistance for resin nanoceramic used in endocrowns but found higher microleakage compared with feldspathic porcelain and lithium disilicate.

Another approach involved using a modified polyetheretherketone (PEEK) framework material veneered with indirect light‐polymerized composite resin. The elastic modulus of PEEK provides better protection to the tooth structure from occlusal forces compared with ceramic materials.^17^

Although ceramic materials offer advantages such as biocompatibility and biomimicry, they may face challenges when subjected to heavy occlusal loads, bruxism, or limited interocclusal space. Ceramic endocrowns can be prone to fracture, especially below the cementoenamel junction.

In the presented case study, the metal endocrown was used as a novel technique to preserve the tooth and avoid extraction or periodontal surgery to gain crown length. It showed promising results during the 5‐year follow‐up period.

In terms of differences in preparation compared with ceramic endocrowns, the metal endocrown utilizes a composite resin base to fill undercuts and ensure the correct design of the preparation. This contributes to significant tissue preservation and acts as a stress breaker.

It is important to note that the information provided is based on the studies and research available in the literature up until September 2021. Further follow‐up studies and advancements in materials and techniques may provide additional insights into the long‐term performance and effectiveness of metal endocrowns.

## 5 SUMMARY

This treatment modality presents a novel technique in restorative dentistry, specifically the use of a metal endocrown as a successful alternative to post‐core crown and a ceramic endocrown. The metal endocrown can be used to preserve the tooth and avoid extraction or periodontal surgery to gain crown length. And it is demonstrated promising results during the 5‐year follow‐up period.

## AUTHOR CONTRIBUTIONS

**Saad AlHazzani:** Investigation; supervision; writing – original draft; writing – review and editing. **Entessar AlAnazi:** Investigation; visualization. **Abdulrhman AlMazam:** Supervision.

## FUNDING INFORMATION

All parts of this presentation were self‐funded.

## CONFLICT OF INTEREST STATEMENT

On behalf of all authors, no conflict of interest is reported.

## ETHICS STATEMENT

Written informed consent was obtained from the patient for the publication of the text, images, and radiographs, and the study was approved by the Departmental Ethical Committee.

## CONSENT

Written informed consent was obtained from the patient to publish this report in accordance with the journal's patient consent policy.

## Data Availability

The authors confirm that the data supporting the presented findings are available within the article.
